# Hydralazine Induced Lupus Syndrome Presenting with Recurrent Pericardial Effusion and a Negative Antinuclear Antibody

**DOI:** 10.1155/2017/5245904

**Published:** 2017-01-17

**Authors:** Praneet Iyer, Ahmed Dirweesh, Ritika Zijoo

**Affiliations:** ^1^Department of Pulmonary and Critical Care, University of Tennessee Health Science Center, Memphis, TN, USA; ^2^Department of Internal Medicine, Seton Hall University School of Health and Medical Sciences, Saint Francis Medical Center, Trenton, NJ, USA

## Abstract

Drug induced lupus erythematosus (DIL or DILE) is an autoimmune disorder caused by chronic use of certain drugs. We report a unique case of hydralazine induced lupus syndrome (HILS) with a negative antinuclear antibody in a female patient who was on hydralazine for a period of 1.5–2 years and developed recurrent pericardial effusion as a result of it. Initially her condition was managed with a pericardial window. The recurrence of a massive pericardial effusion necessitated a right hemipericardiectomy. After hydralazine was stopped, she never had any further episodes of pericardial effusion or tamponade.

## 1. Introduction

Hydralazine induced lupus syndrome (HILS) was first reported in 1953. The syndrome occurs in 5–10% of patients taking hydralazine and clinical manifestations include arthralgia, myalgia, fever, and serositis. In drug induced lupus (DIL) the renal, pulmonary, visceral, and central nervous systems are usually spared. Severe cardiac involvement is rare with only four cases of tamponade previously reported [1–4]. In 95% to 100% of patients with DIL, serum is positive for antinuclear antibody (ANA), which most often has a homogenous pattern. While ANA negative DIL is rare, it has been described [5].

## 2. Case Report

A 36-year-old woman, with past medical history of diabetes, hypertension, hypothyroidism, chronic kidney disease, Lance-Adam syndrome status after cardiopulmonary arrest, and anoxic encephalopathy, presented to our hospital with shortness of breath and chest tightness which started a few days prior to admission. She also complained of orthopnea, paroxysmal nocturnal dyspnea, and productive cough. She had no fever, chills, sick contacts, or recent travel.

The patient denied alcohol and illicit drug abuse. Her prescribed home medications included omeprazole, divalproex, dicyclomine, and numerous antihypertensive medications including hydralazine which was initiated approximately 18 months prior to this admission.

On presentation, vital signs demonstrated a temperature of 98.6 F, respiratory rate of 22 breath/min, blood pressure of 126/102 mmHg, and pulse rate of 92/min.

Pulmonary examination revealed reduced breath sounds at bilateral lung bases. Heart examination revealed normal S1, S2, and S4. Neurological examination showed dysarthria and a left central facial paresis. She had, however, good movement of the upper and lower extremities with intention, severe intentions course action myoclonus in both upper and lower extremities, and hypoactive stretch reflexes.

Significant laboratory findings included hemoglobin of 9 g/dL, creatinine of 2.4 mg/dL (baseline), pro-BNP of 2070 pg/mL, and potassium of 5.5 mmol/L. The rest of the findings were within normal ranges.

Her EKG showed sinus rhythm at 93 beats per minute, prolonged PR interval at 208 ms, and left ventricular hypertrophy, with no changes when compared to prior EKG. Chest radiograph showed severe cardiomegaly with no lung consolidation or pleural abnormality. A transthoracic echocardiogram showed a normal left ventricular function with an EF of 60–65%. There was a moderate to large pericardial effusion with no clear evidence of tamponade. There was mild aortic stenosis noted as well.

The patient had a pericardial window done with drainage of pericardial fluid. Pathological analysis of pericardium showed severe acute and chronic fibrinous and hemorrhagic pericarditis with fibrosis. Cytological analysis of pericardial fluid showed 20% lymphocytes, 65% polymorphonuclear cells, and 15% mesothelial cells present in fresh blood. Pathology and cytology were negative for malignancy and granuloma; special stains for acid fast and fungal organisms were negative. She was then discharged with complete resolution of symptoms.

A follow-up echocardiogram was obtained one week after discharge and demonstrated a small pericardial effusion with no findings to suggest pericardial tamponade and the ejection fraction was 65%.

The patient returned to the emergency department three weeks after with recurrent progressive shortness of breath. Her vitals sign were stable and she was saturating well on room air. Examination demonstrated diminished breath sounds at the left lung base and distant heart sounds. The rest of her physical examination was unchanged from prior admission.

**Figure 1:**
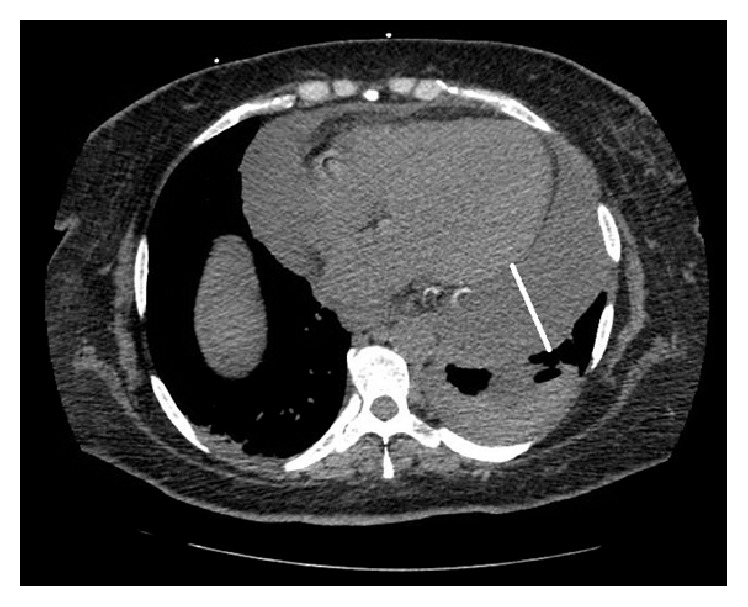
Axial CT chest showing a large pericardial effusion with a small left pleural effusion.

Her chest radiograph showed marked cardiomegaly with prominence of interstitial marking suggestive of congestive changes. CT of the chest without contrast (Figure 1) was performed which showed large pericardial effusion with a small left pleural effusion.

An echocardiogram was performed at bedside which showed large pericardial effusion with evidence of early tamponade physiology.

The patient was admitted to the critical care unit and urgently underwent a left muscle sparing thoracotomy, drainage of left pleural effusion, pericardial resection, and drainage of pericardial effusion.

An echocardiogram was performed one week after this procedure showing no evidence of tamponade, with very small residual pericardial effusion.

As she had developed a recurrent pericardial effusion, an extensive vasculitic and immunological workup was performed. ANA, anti-neutrophil cytoplasmic antibodies (ANCA), anti-double-stranded DNA (ds-DNA) antibody, anti-cyclic citrullinated peptide (CCP) antibody, anti-Smith antibody, ribonuclear protein, SSA antibody, SSB antibody, Scl 70 antibody, and rheumatoid factor were all negative. The anti-histone antibodies were positive.

It was then determined that the patient had recurrent pericardial effusion secondary to drug induced lupus. The instigating drug in this case was hydralazine. Hydralazine was discontinued and the patient was started on colchicine and prednisone which was to be tapered gradually. A repeat echocardiogram 4 months after stopping hydralazine demonstrating complete resolution of pericardial effusion and an ejection fraction of 65–70%.

## 3. Discussion

Since DIL was first described about 50 years ago, more than 70 medications have been implicated as possible etiologic agents. This list includes several antihypertensives of which hydralazine is the most commonly reported medication and poses the most significant risk [6]. The incidence of HILS is dose dependent and is more common in women than men. Approximately, 10.4% of patients on 200 mg or higher dose of hydralazine develop it after at least 3 months of treatment. Cameron and Ramsay reported two patients with pericarditis in their report of several cases of hydralazine induced lupus [7]. After the publication of African American Heart Failure (A-HeFT) trial, there was a significant increase in the amount of hydralazine prescribed to patients with heart failure. Although the dose prescribed to patients during this trial was less than 200 mg daily, evidence now suggests that patients receiving lower doses (<100 mg) are not completely free of risk of hydralazine induced lupus syndrome [8].

Risk factors that have been linked to hydralazine induced lupus include high daily doses (>200 mg), slow acetylator, HLA-DRw4 phenotypes, therapy longer than 3 months, female gender, and a family history of autoimmune disease. HILS is characterized by the following clinical features: arthralgia, fever, anorexia, fatigue, rash, joint pain, and swelling. Musculoskeletal symptoms are the most common clinical manifestation of HILS. It rarely manifests as pericardial effusion, cardiac tamponade, pleural effusion, or pulmonary edema. It was initially thought that renal failure was also uncommon in HILS, but it has been increasingly recognized in some recent studies [8].

Pericardial involvement occurs in <5% of patients with HILS. It usually manifests as pericarditis with or without pericardial effusion. Cardiac tamponade is very rare presentation of HILS and as per our literature search, only four cases have been previously reported. In HILS, pericardial effusion is generally noted to be hemorrhagic [1, 4]. Our patient presented with large hemorrhagic pericardial effusion, while being on 300 mg of hydralazine for a period of 1.5–2 years and had a pericardial window placement during the first admission. She was subsequently discharged and few days later, she returned with recurrent pericardial effusion and tamponade. She subsequently underwent a right sided hemipericardiectomy. After diagnosis of HILS was made, her hydralazine was stopped. Two-dimensional echocardiogram that was done 4 months later did not reveal any evidence of pericardial effusion.

Laboratory findings in HILS include anemia, leukopenia, thrombocytopenia, petechiae, elevated erythrocyte sedimentation (ESR), positive antinuclear antibodies (ANA), and positive anti-histone antibodies. Although a positive ANA titer is used in conjunction with other laboratory tests and clinical findings to confirm the diagnosis of systemic lupus erythematosus, a positive ANA titer alone does not warrant a change in drug therapy because some patients on hydralazine with positive ANA will not have the lupus syndrome. However, ANA is positive in up to 95% with hydralazine induced lupus. Anti-histone antibodies may also be present in up to 95% of the patients with HILS [8]. ANA negative DIL has been rarely reported. Carter et al. reported a case of DIL due to lisinopril with negative ANA, which was the first and only reported case till date [5]. The diagnosis of DIL was made by clinical findings corresponding to lupus and positive anti-histone antibodies. In our patient, ANA was negative and anti-histone antibodies were positive. As per our literature search, this is the second reported case of drug induced lupus and first case of hydralazine induced lupus syndrome with negative ANA and positive anti-histone antibodies.

As mentioned before, the use of hydralazine as an antihypertensive and heart failure medication has increased tremendously in the last decade since the publication of the A-HeFT trial [8]. This was due to an overwhelming 45% relative risk reduction in mortality seen in black patients during the A-HeFT trial [9]. Therefore, more cases of hydralazine induced lupus syndrome have been reported since then as it has been used for longer period of time and at higher doses (>200 mg). Clinicians have to be more vigilant in monitoring for signs of HILS, especially when they are on 200 mg or more of hydralazine for more than 3 months. If our patient had been more closely monitored in the outpatient setting for signs of HILS, then the drug could have been discontinued or switched to another medication sooner and her clinical sequelae of recurrent pericardial effusion or tamponade could have been prevented.

Discontinuation of the offending medication is an integral part of the treatment for drug induced lupus. If symptoms persist after discontinuing the medication or if patient has severe clinical manifestations, then only should corticosteroids or immunosuppressive medications be started. Early diagnosis and treatment are required for critical organ illness in DIL; otherwise associated mortality is high with only supportive care [10].
